# Ethanol effects on ^68^Ga-radiolabelling efficacy and radiolysis in automated synthesis utilizing NaCl post-processing

**DOI:** 10.1186/s41181-019-0076-1

**Published:** 2019-10-07

**Authors:** Michael Meisenheimer, Stefan Kürpig, Markus Essler, Elisabeth Eppard

**Affiliations:** 1 Department of Nuclear Medicine, University Hospital Bonn, Venusberg-Campus 1, Building 21, 53127 Bonn, Germany; 2 PositronPharma S.A, Rancagua 878, 7500921 Providencia, Chile

**Keywords:** Gallium-68, Radiolysis, Radiolabelling yield, Quality control, Ethanol, Module system

## Abstract

**Objective:**

Recent studies showed that ethanol in the reaction mixture improves radiolabelling with trivalent radiometals in terms of precursor amount, reaction time, reaction temperature and radiolysis. With regard to clinical application, this effect is of practical interest in radiopharmacy. The aim of this study was to evaluate whether the positive effect of ethanol can be exploited in automated systems utilizing NaCl-post processing.

**Methods:**

Gallium-68 was obtained from a 1.85 GBq ^68^Ge/^68^Ga-generator. Radiolabelling was performed on an automated ^68^Ga-labelling cassette module. The standard labelling protocol was used without modifications. 0–40 vol% ethanol were added to the reaction mixture. Quality control was performed using radioHPLC and radioTLC.

**Results:**

Utilization of additional ethanol on an automated cassette module can be achieved by adding ethanol directly to the buffer solution without further modifications of the standard procedure. Radiolysis was decreased significantly as analysed by radioHPLC.

**Conclusion:**

It was possible to combine the positive effects of ethanol on radiolabelling efficacy and radiolysis with the standard labelling procedure of an automated cassette module system. The whole process guarantees safe preparation of highly pure ^68^Ga-peptide for clinical application.

## Introduction

One of the most versatile chelators available is the macrocycle 1,4,7,10-tetraazacyclotetradecane 1,4,7,10 tetra acetic acid (DOTA). It is known since 1976 (Stetter and Frank 1976) and was initially used as complexing agent for lanthanides, as it forms stable complexes with most bivalent and trivalent metals (Alexander 1995). In addition, the synthesis of DOTA is very simple and fast, which facilitates the development of many derivatives equipped with various functional groups, which also enable a medical application. The first, and until today used, medical application of DOTA is as the contrast agent gadoteric acid were unfunctionalized DOTA is complexing Gd^3+^ (Caravan et al. 1999).

In nuclear medicine one of the first DOTA derivatives utilized was DOTA-(0)-Phe (1)-Tyr (3))octreotide (DOTA-TOC). It can be applied for both diagnosis, radiolabelled e.g. with gallium-68, or therapy, with e.g. lutetium-177 (Lamberts et al. 1990). The biological active site in DOTA-TOC, TOC, is a somatostatin analogous that binds to somatostatin receptors.

**Fig. 1:**
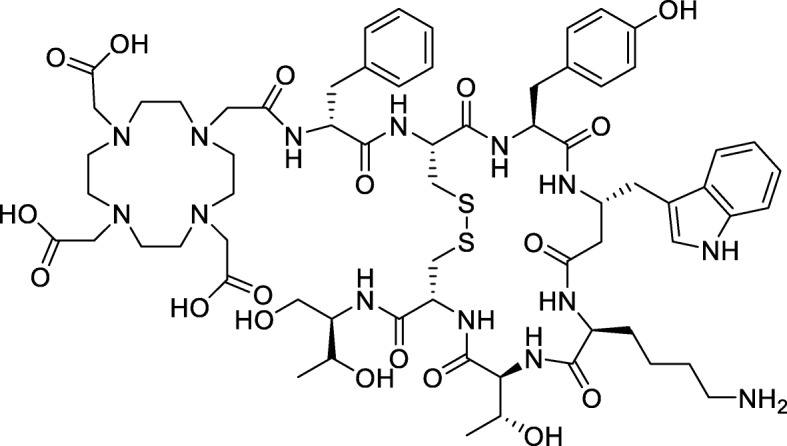
DOTA-conjugated octreotide (DOTA-TOC)

Somatostatin receptors (SSTR) belong to the group of G-protein-coupled receptors. Five subtypes are known (SSTR1-SSTR5), whereby alternative splicing of the SSTR2-mRNA lead to two subtypes, namely SSTR2A and SSTR2B (Prasad et al. 2007). These receptors are overexpressed in a number of neuroendocrine tumours (NET) (Petersenn 2005) why they are well suited as targets for tumour targeting of NETs (Fig. 1).

Radiolabelling of DOTA-TOC can be performed manually or automatically, utilizing a labelling module. Due to the increased radiation exposure for the operator, manual labelling should be avoided in the context of clinical routine where high levels of activity are used. To meet the requirements of radiation protection, radiation exposure should be minimized as far as possible. Correspondingly, automated radiolabelling is preferable to manual synthesis. Besides radiation protection, automation also offers the advantages of a more reliable and repeatable process. Reliability is one of the most important factors in routine production, as quantity and quality of the product must always be guaranteed, which can be ensured by utilizing a module system.

When high-energy particles or ionizing radiation passes through matter, ions and excited molecules are formed (radiolysis products). A number of secondary processes occur following ionization and excitation. Radiolysis cannot be excluded in radiolabelling when high levels of activity are applied. Unfortunately, on the one hand, removal of those radiolysis products is time-consuming and complicated, as well as the verification that they are no longer present in the final product. On the other hand, they can cause undesired and serious side effects when they remain in the final product. Therefore, it is essential to reduce radiolysis by utilizing compounds insensitive to radiation or additives (e.g. radical scavengers) extenuating radiolysis. In clinical context, the applied scavenger should be suitable for human use. Scavengers suitable for human use are ascorbic acid or ethanol. In the proposed labelling process, ethanol is used during the post-processing step. Consequently, it is obvious, to use ethanol as a scavenger. Ethanol is a class 3 solvent that is commonly used as solvent or additive, increasing the solubility of pharmaceuticals or acting as a preservative. Ethanol biocompatible, not showing toxicity or immunoreactivity issues, GMP compatible and has no biological target binding capability (Velikyan 2015). Class 3 solvents may remain indeterminate and unmentioned up to a proportion of 0.5%, but if there is more in a pharmaceutical this must be stated in terms of quantity and identity (European pharmacopeia (9.6. 9.7–9.8) 2019). Previous studies demonstrated the positive influence of ethanol on radiolabelling behaviour and yield as well as the reduction of radiolysis of the product in manual syntheses (Eppard et al. 2017; Helm and Merbach 2005; Mu et al. 2013; Pérez-Malo et al. 2018). Building upon these results, the combination of a more reliable and repeatable method (automation) with a scavenger further improving radiolabelling, should lead to an automated process with a) less peptide needed b) reduced radiolysis by-products c) minimized radiation exposure d) maximized reliability and repeatability. Moreover, all these factors together lead to a cost optimized radiopharmaceutical production.

This work deals with the transfer of ethanol improved radiolabelling to an automated cassette module with prefabricated cassettes and chemical kits with the least possible changes. Therefore, the given process with sodium chlorine post-processing (Mueller et al. 2012) was changed in terms of ethanol content. During the preparation of the cassette, 0–30 vol% ethanol was added to the buffer and synthesis performed without any further changes. Due to reaction volume issues, also buffer modifications were necessary. To obtain comparable results for an ethanol range of 0–40 vol% all experiments were repeated with the new buffer.

## Methods

Gallium-68 was obtained from a ^68^Ge/^68^Ga-generator (iThemba Labs, South-Africa). Synthesis was performed utilizing an automated cassette module (GAIA, Elysia-Raytest, Straubenhardt, Germany). Standard fluidic kit and reagent kit for ^68^Ga-radiolabelling of peptides (ABX advanced biochemical compounds GmbH, Radeberg, Germany) were used. As SCX (strong cation exchanger) 200 mg STRATA SCX (Phenomenex, USA) was used instead of standard SCX included in the reagent kit. TraceSelect water as well as ethanol Ph. Eur. was purchased from Merck (Darmstadt, Germany).

DOTA-TOC was obtained from ABX (ABX advanced biochemical compounds GmbH, Radeberg, Germany), and diluted with TraceSelect water to achieve a final concentration of 1 mg/ml.

DOTA-TATE, obtained from ANASPEC (Fremont, California, USA), was diluted with TraceSelect water to achieve a final concentration of 1 mg/ml.

The standard labelling method provided by the manufacturer: 50 μg DOTA-TOC were labelled with 500 μl post-processed ^68^Ga-eluate in 3.6 ml buffer (0.08 M ammonium acetate buffer, pH 4.5), 8 min, 95 °C. After dilution with ~ 5 ml water subsequent C18 purification of the crude product followed. The product was eluted with 1.5 ml 60 vol% ethanol from the cartridge and finally formulated with 8.5 ml saline and sterile filtered.

The standard labelling method was used with following modifications. 10 μg DOTA-TOC were labelled with 500 μl post-processed ^68^Ga-eluate in 520 μl buffer (0.5 M ammonium acetate buffer, pH 4.5) containing 0–40 vol% ethanol. 8 min, 95 °C. After dilution with ~ 5 ml water subsequent C18 purification of the crude product followed. The product was eluted with 1.5 ml 60 vol% ethanol from the cartridge and finally formulated with 8.5 ml saline and sterile filtered.

For all tests the standard radiolabelling procedure (software method) provided by the manufacturer was used.

All chemicals were of pure or analytical grade and used as received, unless otherwise specified.

For quality control, an aliquot was retained from the final formulation. Quality control was performed with silica-gel coated aluminium TLC-plates (silica 60 F254.5 × 4.5 cm, Merck, Darmstadt, Germany) as well as glass microfiber chromatography paper impregnated with silica-gel (iTLC-SG, Agilent Technologies, Santa Clara, California). Analysis was performed with a single trace radioTLC-scanner (PET-miniGITA, Elysia-Raytest, Straubenhardt, Germany) and evaluation software (GinaStar TLC, Elysia-Raytest, Straubenhardt, Germany). Development of silica TLC-plates was conducted in 0.1 M citrate buffer (pH 4) and 1 M ammonium acetate/methanol (1:1) for iTLC-plates. RadioHPLC was used to determine the radiochemical purity and content of radiolysis products. RadioHPLC was performed using Agilent 1260 Infinity II reverse phase HPLC system (Agilent Technologies, Santa Clara, California) equipped with Gabi γ-HPLC flow detector (Elysia-raytest, Straubenhardt, Germany) and a PC interface running Gina Star software (Elysia-raytest, Straubenhardt, Germany). A Nucleodur 100–3 C18 ec 125/4 column (Macherey-Nagel GmbH & Co. KG, Düren, Germany) was used. The gradient elution system utilized mobile phase A (deionized H_2_O + 0.01% TFA) and mobile phase B (100% acetonitrile + 0.01% TFA) at a flow rate of 0.7 mL/min, starting with 100% A/0% B changing within 20 min to 0% A/100% B, after which gradient parameters are returning to 50% A/50% B during the next 5 min. Radioactivity was measured with a dose calibrator (ISOMED 2010, MED Nuklear-Medizintechnik Dresden GmbH, Dresden, Germany).

**Fig. 2:**
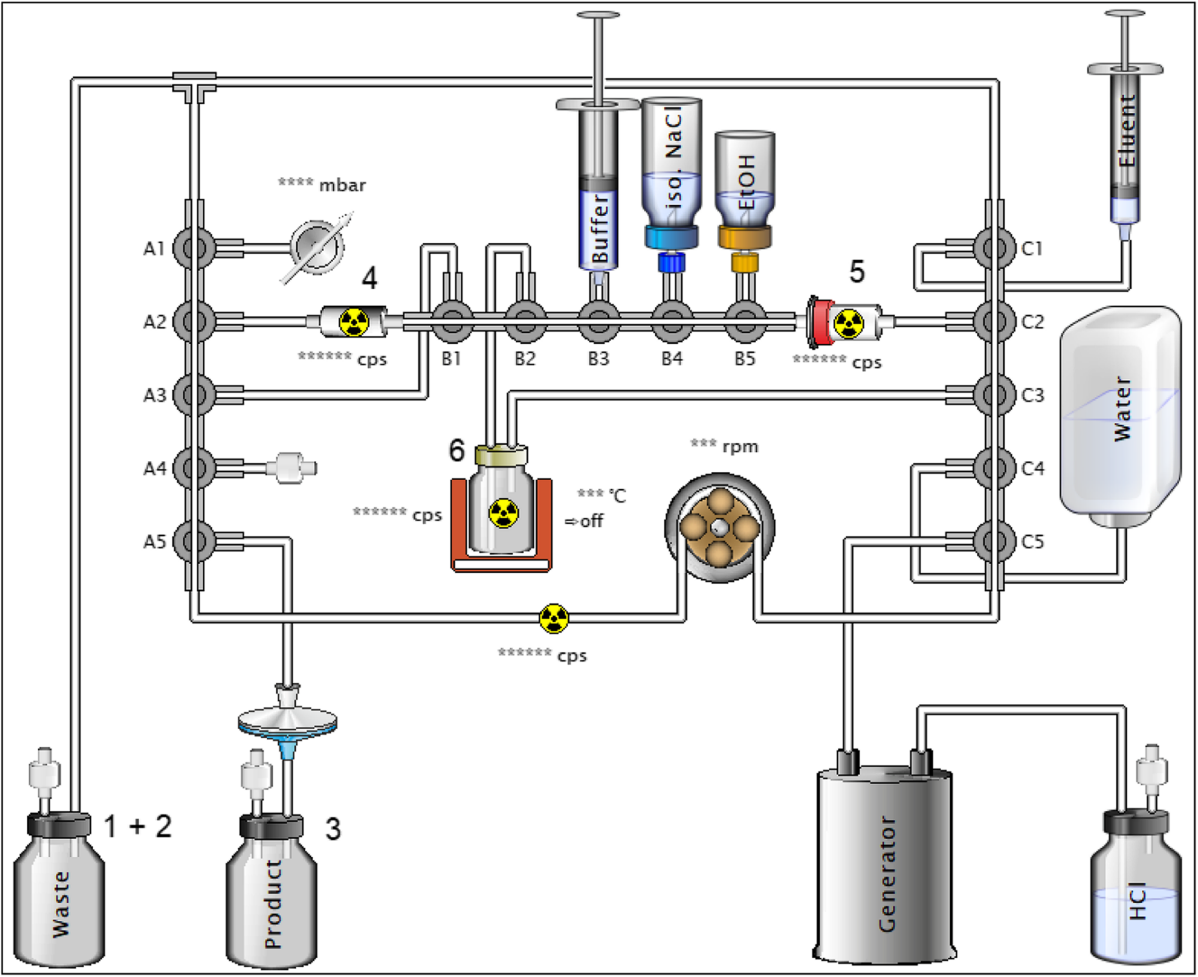
Schematic illustration of the built-in cassette. Visible is the tubing system connecting all three manifolds which has 53-way valves that are operated automatically. Numbers 1–6 indicate radioactivity measurement points

The radiochemical yield was determined based on the decay corrected values of six measuring points (Fig. 2): 1. Waste after generator elution. 2. Waste. 3. Final product. 4. Residual SCX activity. 5. Residual activity of the C18. 6. The reactor. Measuring points 2–6 where measured at the end of the syntheses.

Unless otherwise stated each experiment was performed at least four times to determine the standard deviation.

Statistics were calculated with PRISM Version 8.0.2. All data are expressed as mean ± SD. Groups were compared using the t-test. All statistical tests are two tailed, with a *p*-value of 0.05 representative for significance.

## Results and discussion

The manufacturer of the cassette module recommends the use of 50 μg (35.17 nmol) DOTA-TOC for repeatable and reliable results with guaranteed activity yields > 70%. To be able to determine any effect of ethanol on radiolabelling yields, it was necessary to determine an initial state without ethanol addition, were radiolabelling yields are low enough to measure any effect of ethanol. Systematic reduction of the recommended amount of peptide, confirmed a minimum of 10 μg (7.03 nmol) necessary for reliable automated radiolabelling which was used as initial state.

Starting from this initial state, the content of ethanol in the reaction mixture was increased until 40 vol% were achieved. Due to limited volume of the reactor, it was necessary to modify the buffer with regard of total reaction volume. 3.6 ml buffer with additional 40 vol% ethanol resulted in to an overfull reactor and thus to severe synthesis errors. For this reason, the buffer concentration was increased from 0.08 M (original buffer included in the kit) to 0.5 M. All experiments described used the higher concentrated buffer. Statistical comparison between the results obtained for each buffer showed no statistical significance (Table 1).

**Table 1:** Radiochemical yield compared from DOTA-TOC with 0.08 M buffer and 0.5 M buffer

Vol% ethanol	RCY with 0.08 M buffer [%]	RCY with 0.5 M buffer [%]	*P*-value
0	21.73 ± 7.79	24.38 ± 14.64	0.7601
10	40.09 ± 11.70	42.11 ± 6.99	0.2809
20	75.26 ± 4.59	75.52 ± 2.56	0.0752
30	95.27 ± 3.46	96.25 ± 2.71	0.1284

Since a C18 purification step is included in the synthesis process, radiochemical purity was always ≥98% confirmed by radioTLC and radioHPLC.

Purification via C18 is problematic with organic solvent concentrations higher than 10 vol%. Above this limit, complete trapping of the analyte is not secured. Therefore, manufacturers recommend dilution of the analyte to below 10 vol%. The standard radiolabelling procedure (software method) provided by the manufacturer of the module includes a dilution step before C18 purification. During this step, the reaction mixture is diluted with a total of 5 ml water and cooled down to 40 °C. This reduces the ethanol concentration from 0 to 40 vol% during reaction to 0–5.7 vol%. This concentration is uncritical with regard to C18 purification (Table 2).

**Table 2:** Ethanol content after dilution of the reaction mixture and corresponding activity found in the waste fraction after C-18 trapping as well as on C-18 after elution of the product

Vol% ethanol	Ethanol content after dilution [vol%]	Activity in waste [%]	Activity remaining on C-18 [%]
0	0	55.71 ± 7.73	7.45 ± 6.44
10	0.99	27.51 ± 12.02	15.87 ± 9.88
20	2.21	9.18 ± 1.20	10.18 ± 2,47
30	3.86	0.32 ± 0.08	1.74 ± 0.28
40	5.66	4.27 ± 1.28	3.31 ± 1.58

**Fig. 3:**
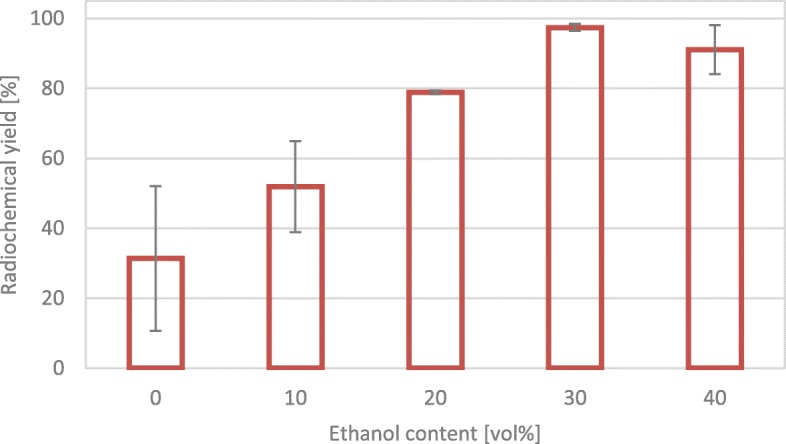
Radiochemical yield depending on ethanol content (vol%) in the reaction mixture. 10 μg (7.03 nmol) DOTA-TOC, T = 95 °C, t = 420 s, 500 μl eluent solution, 0.52 ml 0.5 M ammonium acetate buffer (pH 4.5) for 0–40 vol% ethanol, overall pH 3.6–3.8. All experiments were performed minimum 4 times

Figure 3 shows influence of ethanol on radiochemical yield and reliability of the process at low precursor amounts. Five different concentrations of ethanol were tested with regard to their influence on yield during radiolabelling process. To ensure that the data can be evaluated statistically, all experiments were repeated at least 4 times. As expected, the yield increased from 0 to 30 vol% (Eppard et al. 2017; Pérez-Malo et al. 2018; 2014). The low yield at 0 vol% and 10 vol% is accompanied by a low repeatability expressed in a high standard deviation. One possible factor, which influence the repeatability of the reaction, is the transfer of the peptide in the buffer. Due to the very small amount of peptide already, lowest losses within the syringe and tubing system have a significant effect on radiochemical yields. As the repeatability is decreasing with increasing ethanol content, it can be assumed that the peptide transfer from syringe to reactor via tubing is improved. Actually, ethanol has an influence on solubility of the peptide, thus less peptide adheres to the tubing’s. Accordingly, the losses of peptide during transfer in the tubing system are reduced to a minimum. As a result, even at very low precursor concentrations, the repeatability of the synthesis increases. The radiochemical yield (≥97%) can be compared to a production with 50 μg (35.15 nmol) of DOTA-TOC which is recommended according to supplier’s report.

Figure 3 also shows decreased radiolabelling yield and repeatability for 40 vol% ethanol compared to 30 vol%. This could be explained by the influence of ethanol on the pH value of the reaction (measured) which was not in the optimum range of 3.6–3.8 and by the different behaviour of the ethanol mixtures compared to a pure aqueous solution in the tubing’s. The different behaviour could be explained by the differences in density and viscosity of the two substances and their mixtures. Pure ethanol has a 20% higher viscosity and a density 21% lower than water (Weber 1975). Compared to these results, up to 40 vol% stable and high yields were described in literature (Eppard et al. 2017, 2014). However, these results were obtained in an open vial under constant reaction conditions and without long transfer ways via tubing. Despite of everything no statistical significance was found comparing 30 vol% and 40 vol% ethanol (*p* = 0.0954).

Nevertheless, the effect of ethanol on radiolabelling is significant. Statistical comparison of the different ethanol concentration among each other showed no statistical significance for the increase from 0 vol% to 10 vol% and 30 vol% to 40 vol%. For all other combinations statistical significance is given. Easy adaption to automated synthesis and the considerable increase in radiolabelling yields with only one fifth of precursor amount are two arguments for the use of ethanol in daily routine.

A second beneficial effect of ethanol is its ability to inhibit radiolysis (Velikyan 2015; Mu et al. 2013; Scott et al. 2009). Radiolysis of the precursor occurs as soon as precursor and activity meet each other. The effect of radiolysis inhibition can be shown using radioHPLC.

According to pharmacopoeia, above a value of 0.5 vol% ethanol (European pharmacopeia (9.6. 9.7–9.8) 2019) in the product must be indicated, therefore the maximum possibly quantity present in the final product was calculated. Within this risk assessment, maximum retention of ethanol on the C18 cartridge was supposed.

Before purification, 5 ml water is added to the reactor and then the entire mixture is passed over the C18 cartridge. Afterwards, the reactor is flushed with air until the remaining solution from the C18 has been removed from the reactor. The volume of the remaining solution was determined with a maximum value below 250 μl (*n* ≥ 10).

If this remainder on the C18 cartridge is assumed to be pure ethanol, which would be added to the amount of ethanol used for the purification process, the maximum ethanol content in the final product would be 10.85 vol%.

**Fig. 4:**
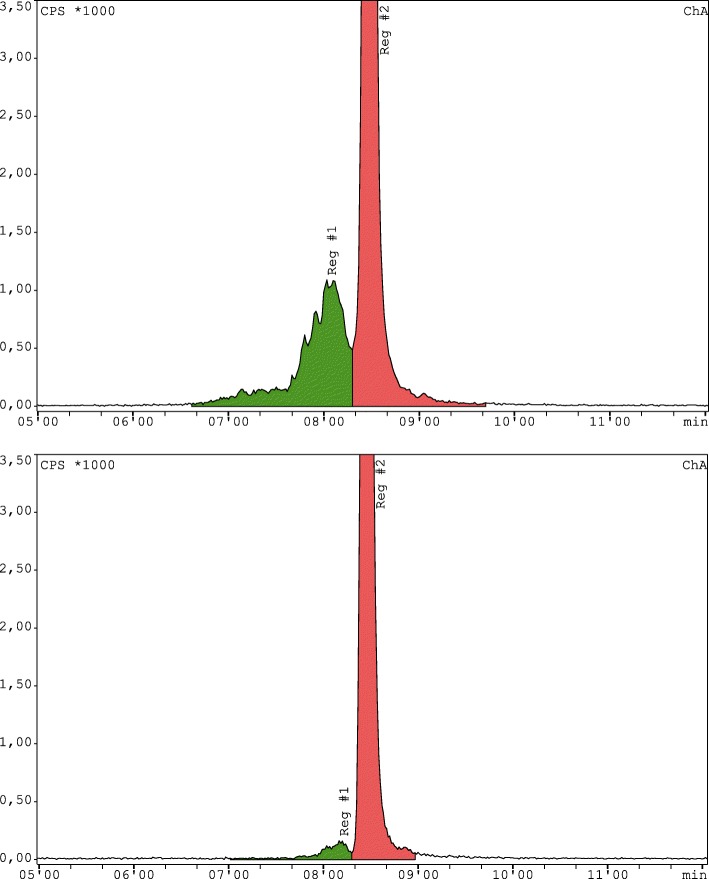
RadioHPLC chromatograms representing a synthesis without (**a**) and with additional ethanol (**b**) in the reaction mixture. Sample collection took place directly after final formulation. In region 1 (8.12 ± 0.13 min) radiolysis by-products can be found. The signal at 8.66 ± 0.08 min in region 2 indicates the product

Figure 4 shows two radioHPLC chromatograms of [^68^Ga]Ga-DOTA-TOC synthesized with (a) and without (b) 5 vol% ethanol in the reaction mixture. To achieve comparable results, in both cases, the quality control sample collection took place directly after final formulation and termination of synthesis. Radiolysis by-products are highlighted in green in the range of 6.7–7.2 min. For simplification it is assumed that by-products detected are formed primarily during the reaction and almost not after the C18 purification step which includes ethanol as eluent.

The amount of radiolysis by-products formed without ethanol as additive in the reaction mixture was 5.87 ± 1.08% (*n* = 150) in comparison to 1.03 ± 0.47% (*n* = 200) utilizing 5 vol% ethanol as radiolysis inhibitor in the reaction mixture. This was found to be statistical significant (*p* < 0.0001).

**Fig. 5:**
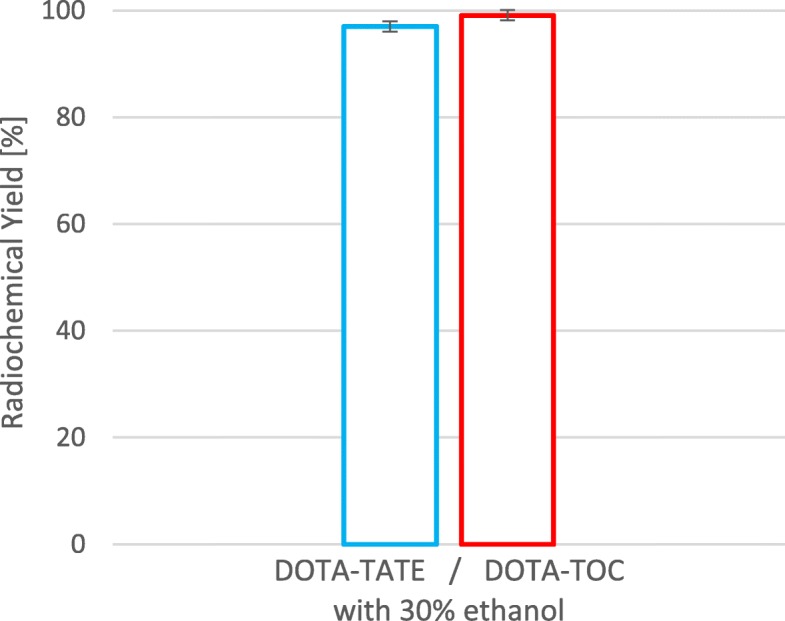
Comparison of the mean radiochemical yield values of DOTA-TATE and DOTA-TOC with 30 vol% ethanol. 10 μg (6.96 nmol) DOTA-TATE or 10 μg (7.03 nmol) DOTA-TOC, T = 95 °C, t = 420 s, 500 μl eluent solution, 0.52 ml 0.5 M ammonium acetate buffer (pH 4), overall pH 3.6–3.8. All experiments were performed minimum 4 times

As the results for the combination of ethanol with an automated process utilizing NaCl post-processing (Mueller et al. 2012) were so clear for DOTA-TOC it was checked, if results can be transferred to another DOTA-conjugated peptide (e.g. DOTA-TATE). Therefore, optimized conditions with 30 vol% ethanol were used for radiolabelling 10 μg (6.96 nmol) DOTA-TATE. In these experiments (*n* = 4) radiochemical yields of 97.00 ± 0.93% and radiochemical purity of ≥98% was obtained. Compared to 10 μg (7.03 nmol) DOTA-TOC with 99.10 ± 0.79% and a radiochemical purity of ≥98%, the results where similar, as expected. Statistical comparison of the results of DOTA-TOC with DOTA-TATE showed no significance (Fig. 5).

## Conclusion

Ethanol can improve an automated cassette based synthesis utilizing NaCl post-processing in terms of precursor amount, repeatability and radiolysis. This can be easily achieved by adding 10 vol% ethanol to the reaction mixture already and leads to optimal results utilizing an ethanol content of 30 vol%.

It is an effective way to decrease the amount of peptide. Reliable and repeatable synthesis can already be achieved with 10 μg (7.03 nmol) of DOTA-TOC utilizing 30 vol% ethanol. This is only one fifth of the precursor amount recommended by the manufacturer of the module system.

Furthermore, it has been shown that radiolysis is already significantly reduced even with only 5 vol% ethanol in the reactor. This is of practical interest especially in clinical daily practise.

## Data Availability

Please contact author for data requests.
